# Immunomodulatory Effects of the *Meretrix Meretrix* Oligopeptide (QLNWD) on Immune-Deficient Mice

**DOI:** 10.3390/molecules24244452

**Published:** 2019-12-05

**Authors:** Wen Zhang, Lei Ye, Fenglei Wang, Jiawen Zheng, Xiaoxiao Tian, Yan Chen, Guofang Ding, Zuisu Yang

**Affiliations:** 1 Zhejiang Provincial Engineering Technology Research Center of Marine Biomedical Products, School of Food and Pharmacy, Zhejiang Ocean University, Zhoushan 316022, China; zhangwen1225z@163.com (W.Z.); Gamma59777@163.com (L.Y.); jwzheng1996@163.com (J.Z.); TIANXIAOXIAO0208@163.com (X.T.); dinggf2007@163.com (G.D.); abc1967@126.com (Z.Y.); 2 Zhejiang Hailisheng Group Co., Ltd., Zhoushan 316021, China; 662199.w@163.com

**Keywords:** *Meretrix meretrix* oligopeptides, cyclophosphamide, immunomodulatory, immune-deficient mice

## Abstract

The aim of this study was to explore the immunomodulatory effects of the *Meretrix meretrix* oligopeptide (MMO, QLNWD) in cyclophosphamide (CTX)-induced immune-deficient mice. Compared to untreated, CTX-induced immune-deficient mice, the spleen and thymus indexes of mice given moderate (100 mg/kg) and high (200 mg/kg) doses of MMO were significantly higher (*p* < 0.05), and body weight loss was alleviated. Hematoxylin-eosin (H&E) staining revealed that MMO reduced spleen injury, thymus injury, and liver injury induced by CTX in mice. Furthermore, MMO boosted the production of immunoglobulin G (IgG) and hemolysin in the serum and promoted the proliferation and differentiation of spleen T-lymphocytes. Taken together, our findings suggest that MMO plays a vital role in protection against immunosuppression in CTX-induced immune-deficient mice and could be a potential immunomodulatory candidate for use in functional foods or immunologic adjuvants.

## 1. Introduction

Immunoregulation can be broadly divided into positive regulation and negative regulation, both of which are the result of complex regulation of the immune system. Sometimes regulation in only one direction is triggered, but most immune regulation is bidirectional in order to maintain a stable steady-state. Immunomodulators can be classified into three general types: immunopotentiators, immunosuppressants, and two-way immunomodulators [1,2,3]. When the body experiences diseases or immune abnormalities, the application of immunomodulators can restore immune function to normal. There are many types of immunomodulators, such as bacterial preparations (e.g., lipopolysaccharide (LPS)), chemical preparations (e.g., cyclophosphamide (CTX)), and biochemical preparations (e.g., thymosin) [4]. However, some chemical immunomodulators have serious side effects, which not only have a specific inhibitory effect on the cause of immune diseases, but also have general inhibitory effects on normal tissue cells [5]. Inflammation, infection, tumors, organ bleeding, and loss of pregnancy have all been reported as being induced after the administration of chemical immunomodulators [6]. Untreated chronic inflammation, however, inhibits natural killer (NK) cells and T cells, which are key participants in the immune system, and limits the success of immunotherapy [7]. More recently, immunomodulators from natural extracts have attracted much attention in the field due to their lesser side-effects when used in humans [8,9]. For example, Hong et al. [10] showed that *Cervus nippon mantchuricus* extract (NGE) has immuno-enhancing effects on RAW264.7 macrophage cells in immunosuppressed mice. Purified leaf extracts of *Melia azedarach* L. (CDM) exerted anti-herpetic activity, inhibited NFκB translocation to the nucleus, and modulated both interleukin (IL)-6 and tumor necrosis factor-alpha (TNF-α) responses in macrophages in one recent study [11]. Therefore, further exploration of natural and effective immunomodulators with lesser side effects seems to be a very worthwhile research pursuit.

Bioactive peptides are small proteins, composed of amino acids, which often have unique physiological functions not possessed by large proteins or their constituent amino acids, such as antibacterial, antiviral, anti-oxidant, antifungal, calcium-binding, or anti-tumor properties [12,13,14]. Moreover, many bioactive peptides can be absorbed and digested even more quickly than free amino acids, and thus have become popular research topics and promising functional factors in the international food industry [15]. As the most common kind of bioactive peptide, immunologically active peptides stimulate the proliferation of lymphocytes, enhance the phagocytic abilities of macrophages, improve the body’s resistance to external pathogens, and generally enhance the body’s immunity to infection. Recently, such immunoregulatory peptides have attracted much research attention. For example, Yang et al. [16] reported that a marine oligopeptide from chum salmon could significantly enhance the capacity of lymphocyte proliferation in mice. Gao et al. [17] reported that collagen hydrolysates from yak bones exhibited immunomodulatory effects on CTX-induced immunosuppressed mice by increasing both innate and adaptive immunity. Li et al. [18] reported that a novel pentapeptide (RVAPEEHPVEGRYLV) from *Cyclina sinensis* could stimulate macrophage activity to activate the NFκB signaling pathway, and further in vivo studies revealed that this novel pentapeptide has immunomodulatory effects on CTX-induced immunosuppression in mice [19].

In a previous study of ours, an oligopeptide (QLNWD) was purified from the hydrolysate of *Meretrix meretrix* oligopeptide (MMO), and was shown to have the ability to aid in reversing the effects of nonalcoholic fatty liver disease (NAFLD) in mice [20]. We also investigated the immunomodulatory effect of this oligopeptide in vitro [21], and our results indicated that MMO has the effect of promoting the activation of RAW264.7 cells and the potential to enhance the non-specific immunity. However, the immunoregulatory activity of this oligopeptide in vivo is unknown. The aim of the present study was to explore the immunomodulatory effects of MMO on mice with CTX-induced immunosuppression in vivo. The effects of MMO on the thymus and spleen indexes of the mice were investigated, as well as morphological changes to their spleens, thymuses, and livers as observed microscopically using hematoxylin-eosin (H&E) staining. The stimulation index change in spleen T-lymphocytes was also determined in the present study. This study will provide a foundation for the further development of MMO as an immunopotentiator.

## 2. Results and Discussion

### 2.1. Comparison of Body Weight

**Figure 1:**
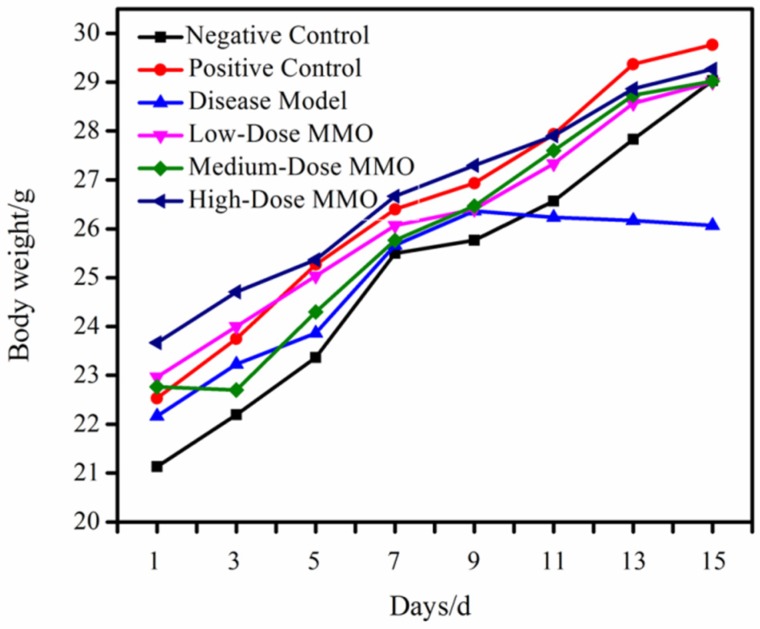
The body weight changes of immunosuppressed study mice. Negative control: saline; positive control: 25 mg/kg of levamisole; disease model: 80 mg/kg of CTX; low-dose MMO: 50 mg/kg of MMO; medium-dose MMO: 100 mg/kg of MMO; and high-dose MMO: 200 mg/kg of MMO.

The body weight of mice is a direct indicator of their physical condition. Previous evidence has shown that weight recovery can effectively increase the number of T-cell subsets and macrophages, which are vital components of the murine immune system [22,23,24]. As shown in Figure 1, it was observed that the weights of the mice in the positive drug or MMO-treated groups were significantly reduced when compared to the control group in the five days prior to the commencement of the study. Over the next 10 days, the mice in both the positive control group and the MMO-treated group saw marked increases in their body weights. However, the body weight of mice in the disease model became stable after 7 days, as it seems that CTX can cause immunodeficiency in mice, resulting in reduced appetite. This result indicated that MMO had an effect on alleviating the degree of immunosuppression induced by CTX on the mice.

### 2.2. Thymus and Spleen Indexes

**Figure 2:**
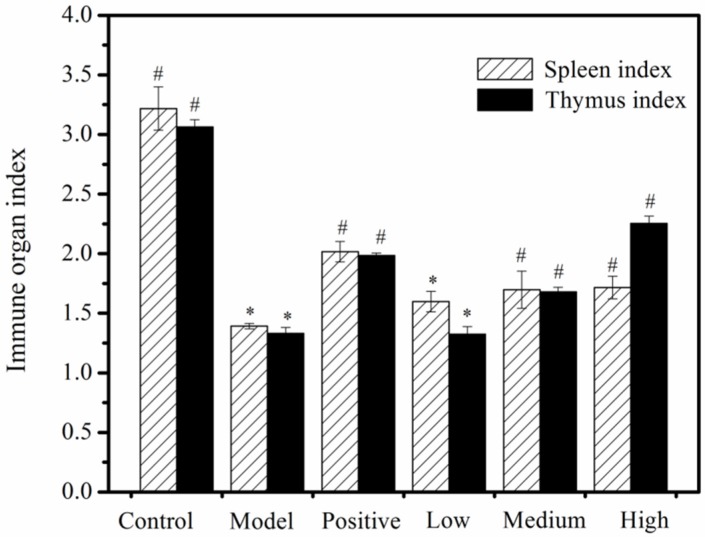
MMO-induced changes to the immune organ indexes of mice. *, a significant difference when compared to the negative control (*p* < 0.05); ^#^, a significant difference when compared to the disease model, *p* < 0.05 values were considered to be statistically significant.

The thymus and spleen are representative immune organs. The thymus is one of the primary lymphoid organs [25], and the innate and adaptive immune responses to antigens and pathogens are initiated by the spleen, which is considered to be an important organ for assessing immune system function [26]. The thymus and spleen indexes can thus be used to roughly estimate the strength of immune function, which is a superficial and lagging indicator [27]. Our results showed that the spleen and thymus indexes of the model group were visibly reduced (*p* < 0.05) when compared to the negative control group. The spleen indexes of the middle (100 mg/kg) and high (200 mg/kg) MMO groups were significantly higher than those of the model group (*p* < 0.05), which was similar for their thymus indexes as well (Figure 2). These results indicate that MMO may be able to effectively alleviate the atrophy of both the spleen and the thymus caused by CTX.

### 2.3. Morphological Observations of Mouse Organs

**Figure 3:**
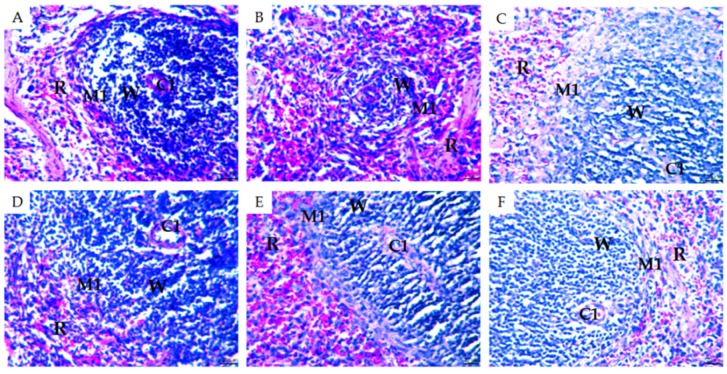
H&E staining of mouse spleen (×400). R: red medulla; W: white medulla; M1: marginal area; C1: central artery. (**A**) Negative control; (**B**) disease model; (**C**) positive control; (**D**) low-dose (50 mg/kg) MMO; (**E**) medium-dose (100 mg/kg) MMO; and (**F**) high-dose (200 mg/kg) MMO.

**Figure 4:**
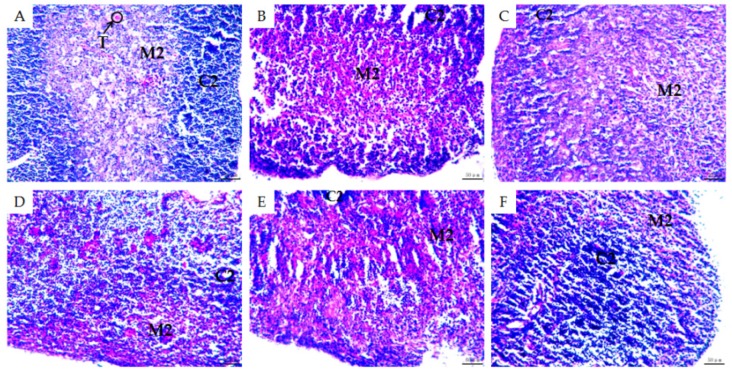
H&E staining of mice thymus (×200). C2: cortical; M2: medulla; T: thymus corpuscle. (**A**) Negative control; (**B**) disease model; (**C**) positive control; (**D**) low-dose (50 mg/kg) MMO; (**E**) medium-dose (100 mg/kg) MMO; and (**F**) high-dose (200 mg/kg) MMO.

**Figure 5:**
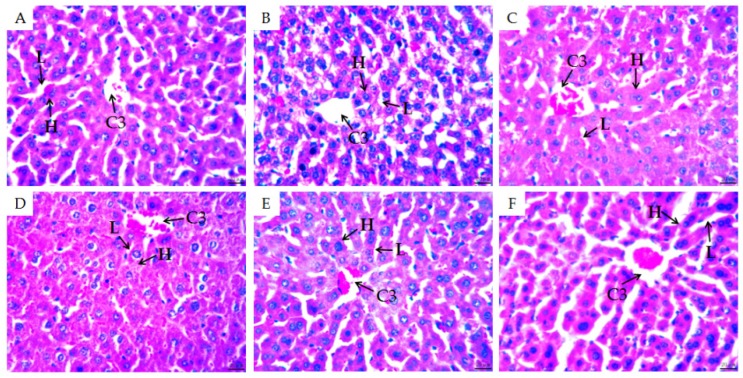
H&E staining of mice livers (×400). C3: central vein; H: hepatic cord; L: liver sinusoidal. (**A**) Negative control; (**B**) disease model group; (**C**) positive control; (**D**) low-dose (50 mg/kg) MMO; (**E**) medium-dose (100 mg/kg) MMO; and (**F**) high-dose (200 mg/kg) MMO.

To gain further insight into the effectiveness of MMO on CTX-induced immunosuppression in the mice, H&E staining was used to observe subtle morphological changes to the spleens, thymus glands, and livers of the mice in each group (Figure 3, Figure 4 and Figure 5).

The spleen is the largest immune organ of the body, accounting for 25% of the total lymphoid tissue, and also contains a large number of lymphocytes, dendritic cells, neutrophils, natural killer cells, and macrophages [28]. As shown in Figure 3, there was a clear dividing line between the red and white medulla in the normal group (Figure 3A). The splenic corpuscle in the white medulla was nearly round, the lymphatic sheath structure around the artery was complete, the splenic cord in the red medulla was connected, and the splenic sinus was obvious. In the model group, the boundary between the white pulp and the red pulp was blurred and the splenic corpuscle in the white pulp was scattered (Figure 3B). Compared to the normal group, the lymphatic sheath around the artery was thinner and the area of the red pulp was smaller, which suggested that CTX may have damaged the T and B cells in the spleen, significantly reduced the lymphoid tissue, and led to overall atrophy of the spleen. In the positive control group, multiple intact splenic corpuscles were seen, with an enlarged white medullary margin and thickened lymphatic sheath around the central artery (Figure 3C). The general structure of the splenic corpuscle was observed in the low-dose MMO group, but the boundary between the red and white medulla was still not obvious (Figure 3D). In the medium-dose MMO group, the marginal area of white pulp could be observed (Figure 3E). Red and white medulla were clearly observed in the high-dose MMO group and the white medulla margins were widened (Figure 3F). Overall, MMO gradually returned the spleen structure to an organizational form similar to that of the normal group. The white pulp part, for example, became clearer and, for the high-dose MMO group in particular, presented with a shape very similar to that of the positive control group. These results suggest that MMO can restore lymphocyte white marrow, increase T and B cells in marginal regions, and reduce CTX-induced spleen cell apoptosis in mice [29].

Thymus atrophy also showed a similar trend in all of the mice. The cortex of the thymus contains thymocytes, which produce thymosin that can stimulate the proliferation and differentiation of T-lymphocytes, activate the major histocompatibility complex (MHC) colony factor transmitting signal [30], and accelerate the presentation of antigens. In the normal group, cortical and medullary structures were clear and distinct and obvious thymus bodies were observed in the medulla (Figure 4A). In the model group, the cortex and medulla were intercalated, the thymus corpuscle was shrunken and unclear in the visual field, the cortical area was smaller, and the number of T-lymphocytes was significantly reduced (Figure 4B), which all indicate significant immunosuppression when compared to the normal group. Cells and T-lymphocytes in the thymus of the positive control group were significantly increased when compared to the negative control group and multiple thymus corpuscles were observed in the visual field (Figure 4C). The cortex and medulla of the low-dose MMO group still could not be distinguished, but there was an increase in T cells in the cortex (Figure 4D). In the medium-dose MMO group, the cortex and medulla could be distinguished only roughly (Figure 4E). In the high-dose MMO group, however, the cortex and medulla were distinct and T-lymphocytes were significantly increased (Figure 4F)—a similar morphology to that of the positive control group. With increasing doses of MMO, cortical thymocytes increased, which demonstrated that MMO could activate the immune response and reduce the thymus injury induced by CTX [30].

The liver is the central hub of the body’s metabolism, with functions such as detoxification and hematopoiesis [31]. To investigate whether there was an effect on the liver after using immunosuppressive agents and MMO, the histological structure of the mouse liver was observed. The hepatic lobule structure of the negative control group was clear and complete, with radial hepatic cords that radiated out in all directions and were arranged neatly around the central vein. Morphology of the hepatocytes was also regular and liver sinusoidal structures were observed (Figure 5A). In the disease model group, the hepatic cord was ruptured and disordered and degeneration and necrosis of hepatocytes was evidenced by a reduction in vacuoles and even absence of part of the nucleus. The structure of the liver sinusoids was not obvious, indicating that the cytotoxicity of CTX caused damage to the mouse liver (Figure 5B). In the positive control group, the hepatic cord around the central vein recovered to the radial structure and the hepatocyte nucleus also showed a round shape (Figure 5C). The liver morphologies of the medium-dose and the high-dose MMO groups both bore a close resemblance to that of the positive control group (Figure 5E,F). Our results showed that MMO can effectively reduce the cytotoxicity of CTX-induced liver injury in mice.

### 2.4. Serum Immunoglobulin G (IgG) Levels

**Figure 6:**
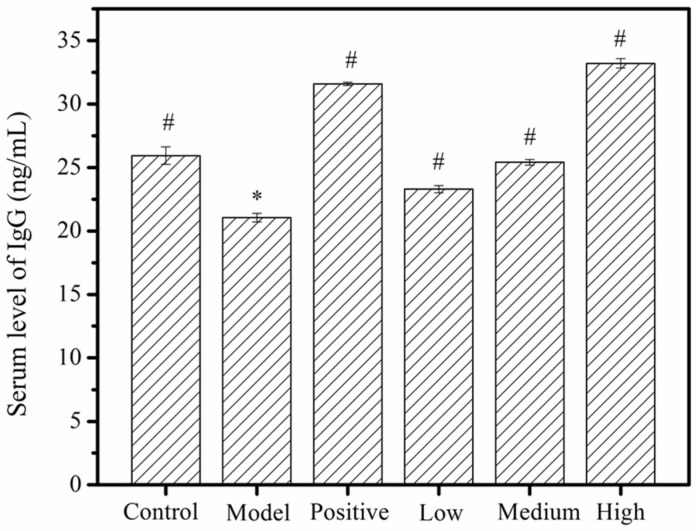
The effects of MMO on immunoglobulin G (IgG) content in mouse serum. *, a significant difference when compared to the negative control, *p* < 0.05. ^#^, a significant difference when compared to the disease model, *p* < 0.05. *p* < 0.05 values were considered to be statistically significant.

IgG is one of the most abundant proteins in human serum, accounting for about 10–20% of plasma protein [32]. Detection of IgG levels can help to indirectly judge the immune function of the body [33]. Vikas et al. [34] explored the changes in IgG levels in mice treated with galactose. The results showed that the IgG concentration in the galactose-treated mice was higher than that of the normal group, indicating that galactose had the potential to upregulate IgG production. The effect of MMO on IgG content in mice serum is shown in Figure 6. The IgG level in the model group was markedly reduced when compared to the negative control group (*p* < 0.05). From the doses of 50 mg/kg to 200 mg/kg, the MMO-treated groups seemed to have significant dose-dependent increases in IgG levels (*p* < 0.05), when compared to the disease model group. Moreover, the IgG levels in the high-dose MMO group were higher than the negative control and close to the positive control group, which suggests that high doses of MMO have a very beneficial effect on the restoration of serum immunoglobulins in immunocompromised mice.

### 2.5. Serum Hemolysin

Hemolysin reflects the proliferation and differentiation of hemolytic B cells and is one of the main nonspecific indexes used to measure the immune function of the body [35,36]. The half hemolysis value (HC_50_) and the hemolysin proliferation rate are routinely used to evaluate the effects of natural extract products on humoral immunity in mice [36]. As shown in Table 1, in contrast to the negative control group, the HC_50_ of the model group dropped by 0.68 ± 0.05. However, the HC_50_ levels of the MMO-treated groups (50, 100, 200 mg/kg) were raised by 0.26 ± 0.05, 1.69 ± 0.02, and 3.20 ± 0.05, respectively. The proliferation rate of hemolysin in the disease model group was −0.78% ± 0.05 when compared to the negative control group, which indicated that serum hemolysin was inhibited by CTX. However, the proliferation rates of the MMO-treated groups exceeded that of the negative control group across the board, suggesting a supra-accelerating effect of MMO on CTX-damaged mice. Similarly, Pan et al. [35] reported that milk protein hydrolysate (MPH) increased immunological function by triggering hemolysin formation in mice. Liu et al. [37] showed that cottonseed meal oligopeptide (PFC) significantly increased the HC_50_ levels in mice by 1.39 ± 0.45, 2.59 ± 0.20, and 2.46 ± 0.41 when given doses of 5 mg/mL, 10 mg/mL, and 20 mg/mL, respectively. Our results were consistent with these findings and indicated that MMO has the effect of alleviating immunosuppression induced by CTX in mice.

### 2.6. T Lymphocyte Assessment

**Figure 7:**
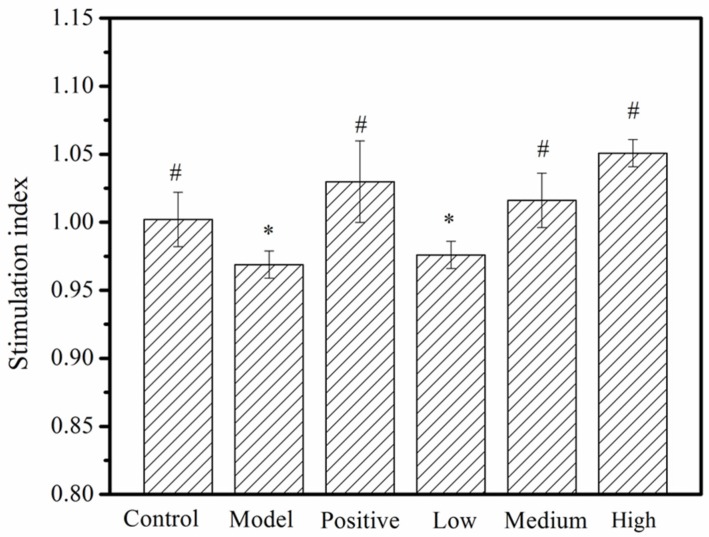
The stimulation index change induced by MMO in spleen T-lymphocytes from mice. *, a significant difference when compared to the negative control (*p* < 0.05); ^#^, a significant difference when compared to the disease model (*p* < 0.05). *p* < 0.05 values were considered to be statistically significant.

As the main cells in both the thymus and the spleen, T-lymphocytes can assist B cells to produce antibodies, kill target cells, and promote mitogen responses [38,39]. Relevant studies have reported that mitogens, such as Concanavalin A (ConA) and phytohemagglutinin (PHA), can stimulate lymphocytes to release a wide variety of cytokines in vitro as well as induce the simultaneous stimulatory and inhibitory activities of different T cell populations [40]. Evidence has shown that one effect of ConA stimulation of T-lymphocytes may be to enhance endocytosis of the cell membrane and studies have speculated that cell density and cell contact area are associated with the stimulation of ConA, reaching a peak of growth at 24 h [41,42]. In this study, we used ConA to stimulate spleen T-lymphocytes extracted from the spleens of each group of mice and observed the stimulation effects after 24 h. We observed that the stimulation of T-lymphocytes in the model group was lower than in the negative control, which indicated that CTX inhibited the responses of T cells in the lymphatic systems of those mice. The net proliferation of T-lymphocytes in the low-dose (50 mg/kg) MMO group was similar to that of the model group. However, the net proliferation of T-lymphocytes in the high-dose MMO-treated group was higher than that of the negative control group, indicating that MMO stimulated the proliferation of T-lymphocytes and reduced the inhibitory effects of CTX on T-lymphocytes in those mice (Figure 7). Combined with the H&E staining results, we speculated that spleen atrophy was reduced by MMO and that the activity of T-lymphocytes was increased in CTX-immunocompromised mice.

## 3. Materials and Methods

### 3.1. Animals

Sixty male ICR mice (20–23 g) were provided by the Experiment Animal Center of Zhejiang Province (certificate no SCXK 2014-0001). All the mice were kept under conventional and uniform conditions at 22 °C. The study proceeded after the mice were given seven days to acclimatize to their new environment.

### 3.2. Materials and Chemical Reagents

The MMO (QLNWD) [13] used for the experiments was synthesized by China Peptides Co., Ltd. (Shanghai, China). The CTX and levamisole were provided by Shanghai Yuanye Bio-Technology Co., Ltd. (Shanghai, China). An H&E staining kit was supplied by Nanjing Jiancheng Bioengineering Institute (Jiangsu, China). Sheep red blood cells (SRBC) and guinea pig serum were obtained from Zhengzhou Baiji Biological Engineering Co. Ltd. (Henan, China). A mouse IgG enzyme linked immunosorbent assay (ELISA) kit was purchased from Shanghai Fusheng Industrial Co. Ltd. (Shanghai, China). Hanks’ balanced salt solution (HBSS) and ConA were purchased from Solarbio (Beijing, China). NLRP3 rabbit monoclonal antibody was purchased from Cell Signaling Technology (Massachusetts, USA). A 3,3′-diaminobenzidine (DAB) immunohistochemistry color development kit was purchased from BBI Life Science Corporation Co., Ltd. (Shanghai, China). Ammonium-chloride-potassium (ACK) lysis buffer was offered by Beyotime Biotechnology (Shanghai, China).

### 3.3. Animal Groupings and Treatments

Animal groupings and procedures were performed according to the methods in Zhang et al. [43] with some slight modifications. The mice were randomly divided into 6 groups, each of which contained 10 mice. Each mouse had its body weight recorded, received an intraperitoneal injection of 0.2 mL, and was fed the same weight of feed every day at the same time. The experimental groups were administered three dosage concentrations of MMO: 50 mg/kg body weight (BW) (low dose), 100 mg/kg BW (medium dose), and 200 mg/kg BW (high dose). The negative control group and the disease model group were given normal saline (NS, 0.9% NaCl) injections. The positive control group was protected from the effects of CTX by 2.5 mg/kg BW of levamisole given over 10 days prior to the experiment [44]. On day 1 of the experiment, all groups except the negative control group were injected with 80 mg/kg BW CTX (Table 2, after having fasted without water deprivation for 24 h beforehand [45].

### 3.4. Body Weight and Immune Organ Index Changes

The body weights of all mice were recorded once every other day for 15 days total. Before being sacrificed by cervical dislocation, each mouse was weighed a final time. The immune organs and the spleen and thymus glands were harvested, rinsed using NS, blotted by gauze immediately, and weighed in order to calculate each mouse’s immune organ index (IOI) using Equation (1), before finally proceeding to dissection:

(1) $$\mathrm{IOI}=\frac{\mathrm{immune}\;\mathrm{organ}\;\mathrm{weight}}{\mathrm{bodyweight}}\times 100\%.$$

### 3.5. Histomorphological Observation

Following dissection, the tissues were fixed in 4% paraformaldehyde for 24 h to 48 h, embedded in paraffin, sliced to 5 μm sections, stained using an H&E staining kit, and sealed with neutral gum. The histomorphological changes of the organ tissues of each group were observed under an optical microscope (CX31, Olympus) and photographed with a CCD-NC 6051 photographic system.

### 3.6. Determination of IgG Serum Content

Blood was sampled from the eyes 24 h after each mouse’s last intraperitoneal injection. The serum and plasma were separated using a refrigerated centrifuge (4 °C, 5000 rpm, 5 min). The amount of IgG in the serum was measured by a mouse IgG ELISA kit from Shanghai Fusheng Industrial Co. Ltd.

### 3.7. Detection of Serum Hemolysin

The mice serum samples were diluted 100-fold in 96-well plates at 100 μL per well. The sample wells were mixed with 5% sheep red blood cells (SRBC) (50 μL) and 10% guinea pig serum (50 μL), while control wells had just 5% SRBC (50 μL) added to them. The 96-well plates were placed in a 37 °C water bath for 30 min, after which the reaction was stopped in ice water and the supernatants were collected and analyzed at 540 nm in a microplate reader (SpectraMax M2, Molecular Devices, San Jose, CA, USA). The half hemolysis value (HC_50_) and the hemolysin content change showed the change of hemolysin in the serum samples of the mice (Equations (2) and (3)).

(2) $${\mathrm{HC}}_{50}=\frac{\mathrm{OD}\;\mathrm{value}\;\mathrm{of}\;\mathrm{sample}\times \mathrm{dilution}\;\mathrm{ratio}}{\mathrm{OD}\;\mathrm{value}\;\mathrm{of}\;\mathrm{SRBC}},$$

(3) $$\mathrm{Proliferation}\;\mathrm{rate}=\frac{\left({\mathrm{HC}}_{50}\;\mathrm{of}\;\mathrm{sample}-{\mathrm{HC}}_{50}\;\mathrm{of}\;\mathrm{control}\right)}{{\mathrm{HC}}_{50}\;\mathrm{of}\;\mathrm{control}}.$$

### 3.8. Proliferation of Spleen T-Lymphocytes

Mice spleen T-lymphocytes were extracted by the method described by Cai et al. [46]. The spleens of the mice were carefully dissected on a sterile bench, washed with HBSS, cleaned of blood and unrelated tissues, and ground on a 200-mesh stainless steel mesh, after which the cells were collected in a clean centrifuge tube. After being centrifuged at 1000 rpm for 5 min, the supernatant was discarded and the pellet was mixed with 2 mL of ammonium-chloride-potassium (ACK) lysis buffer for 5 min, washing three times with HBSS in between each step. After centrifugation under uniform conditions, the remaining cells were resuspended in RPMI-1640 complete medium, cultured in a cell culture incubator for 12 h, and stored for subsequent multiplex reaction experiments.

The T lymphocyte multiplication experiment method described by Ye et al. [47] was adopted for this study as well. The pre-preparation cell suspension was then put into a 96-well plate and the number of cells was 1 × 10^6^ cells/mL. Each concentration was set into four complex wells (200 μL). Then, 10 μL of ConA (5 μg/mL) was added among 2 wells and 10 μL NS added as control. The plate was incubated in an incubator (37 °C, 5% CO_2_) for 24 h, to which was added 3-(4,5-dimethyl-2-thiazolyl)-2,5-diphenyl-2-*H*-tetrazolium bromide (MTT) during the 20 h. Finally, it was treated with 150 μL DMSO for 10 min and the absorbance was detected under 490 nm. The T lymphocyte-multiplication extent was represented by the stimulation index (SI), calculated as shown below Equation (4):

(4) $$\mathrm{SI}=\frac{\mathrm{OD}\;\mathrm{value}\;\mathrm{of}\;\mathrm{sample}\;\mathrm{well}\;\left(\mathrm{average}\right)}{\mathrm{OD}\;\mathrm{value}\;\mathrm{of}\;\mathrm{control}\;\left(\mathrm{average}\right)}$$

### 3.9. Statistical Analysis

The experimental data were analyzed and processed by SPSS 19.0 statistical software. The figures were expressed as mean ± standard deviation (SD), analyzed using a one-way analysis of variance (ANOVA) test, and *p* < 0.05 values were considered to be statistically significant.

## 4. Conclusions

In general, we have conclusively shown that MMO has immunomodulatory effects on CTX-immunocompromised mice. Compared to the disease model group, 100 mg/kg and 200 mg/kg doses of MMO were shown to significantly increase the spleen and thymus indexes (*p* < 0.05) and alleviate CTX-induced body weight loss in our experimental mice. The spleen immune injuries and thymus injuries induced by CTX were also alleviated in the MMO-treated groups. Furthermore, MMO may increase the levels of IgG and hemolysin in mouse serum and promote the proliferation of spleen T-lymphocytes. Our findings suggest that MMO plays a vital role in protection against immunosuppression in CTX-treated mice. Transcriptomics and proteomics will be used to further reveal its immune regulatory mechanism in our future studies in vitro and in vivo. We hope that our findings will provide a foundation for further study of MMO as an immunoregulatory adjuvant or functional food additive.

## Figures and Tables

**Table 1:** The effect of MMO on HC_50_ and the hemolysin proliferation rate.

Group	HC_50_	Proliferation Rate %
Negative Control	87.58 ± 0.05	0
Diseased Model	86.90 ± 0.05	−0.78 ± 0.05
Positive Control	91.39 ± 0.03 *	4.35 ± 0.03 *
Low-dose	87.84 ± 0.10	0.29 ± 0.10
Medium-dose	89.27 ± 0.07	1.92 ± 0.07
High-dose	90.78 ± 0.05 *	3.65 ± 0.05 *

Note: * indicates a significant difference over the Negative Control group.

**Table 2:** Mice groupings and treatments.

Group	Pre-Treatment (10 days)	Treatment (5 days)
	Dose (0.2 mL)	Dose (0.2 mL)
Negative Control	NS	NS
Disease Model	NS	CTX (80 mg/kg BW)
Positive Control	Levamisole (25 mg/kg BW)	CTX (80 mg/kg BW)
Low Dose	MMO (50 mg/kg BW)	CTX (80 mg/kg BW)
Medium Dose	MMO (100 mg/kg BW)	CTX (80 mg/kg BW)
High Dose	MMO (200 mg/kg BW)	CTX (80 mg/kg BW)
